# PARP Inhibitors in Metastatic Castration-Resistant Prostate Cancer: Unraveling the Therapeutic Landscape

**DOI:** 10.3390/life14020198

**Published:** 2024-01-30

**Authors:** Ashaar Al-Akhras, Chadi Hage Chehade, Arshit Narang, Umang Swami

**Affiliations:** 1 Faculty of Medicine, Jordan University of Science and Technology, Irbid 22110, Jordan; analakhras19@med.just.edu.jo; 2 Division of Medical Oncology, Department of Internal Medicine, Huntsman Cancer Institute, University of Utah, Salt Lake City, UT 84112, USA; chadi.chehade@hci.utah.edu (C.H.C.); arshit.narang@hci.utah.edu (A.N.)

**Keywords:** metastatic prostate cancer, poly-ADP ribose polymerase inhibitors, BRCA, HRR

## Abstract

The treatment landscape of metastatic prostate cancer (mPCa) is rapidly evolving with the recent approvals of poly-ADP ribose polymerase inhibitors (PARPis) as monotherapy or as part of combination therapy with androgen receptor pathway inhibitors in patients with metastatic castration-resistant prostate cancer (mCRPC). Already part of the therapeutic armamentarium in different types of advanced cancers, these molecules have shaped a new era in mPCa by targeting genomic pathways altered in these patients, leading to promising responses. These agents act by inhibiting poly-ADP ribose polymerase (PARP) enzymes involved in repairing single-strand breaks in the DNA. Based on the PROfound and TRITON3 trials, olaparib and rucaparib were respectively approved as monotherapy in pretreated patients with mCRPC and alterations in prespecified genes. The combinations of olaparib with abiraterone (PROpel) and niraparib with abiraterone (MAGNITUDE) were approved as first-line options in patients with mCRPC and alterations in *BRCA1/2*, whereas the combination of talazoparib with enzalutamide (TALAPRO-2) was approved in the same setting in patients with alterations in any of the HRR genes, which are found in around a quarter of patients with advanced prostate cancer. Additional trials are already underway to assess these agents in an earlier hormone-sensitive setting. Future directions will include refining the treatment sequencing in patients with mCRPC in the clinic while taking into account the financial toxicity as well as the potential side effects encountered with these therapies and elucidating their mechanism of action in patients with non-altered HRR genes. Herein, we review the biological rationale behind using PARPis in mCRPC and the key aforementioned clinical trials that paved the way for these approvals.

## 1. Introduction

Prostate cancer (PCa) is the most common non-cutaneous malignant neoplasm in men, accounting for 29% of new cancer diagnoses, and the second leading cause of cancer-related death [1,2]. The 5-year survival rate of patients with metastatic PCa (mPCa) remains low at about 32% despite advances in treatment regimens and strategies in the last decade [3,4]. 

Genomic instability is one of the hallmarks of cancer and is commonly caused by defective DNA damage repair pathways, including mutations in homologous recombination repair (HRR) genes such as BReast CAncer gene 1/gene 2 (*BRCA1/2*) [5]. The prevalence of germline and somatic HRR mutations reaches 12% and 20-25%, respectively, in patients with metastatic castration-resistant prostate cancer (mCRPC) [6], exceeding their frequency in localized PCa of 3.5% and 8%, respectively [7,8]. The most frequently mutated HRR gene in mCRPC is *BRCA2* (44%), followed by *ATM*, *CHEK2*, and *BRCA1,* which account for 13%, 12%, and 7% of HRR mutations in patients with mCRPC, respectively [9]. It is important to note that these patients have poor survival outcomes with characteristically more aggressive and poorly differentiated disease, stressing the need for more specialized therapeutic approaches in this patient subset [10].

**Figure 1:**
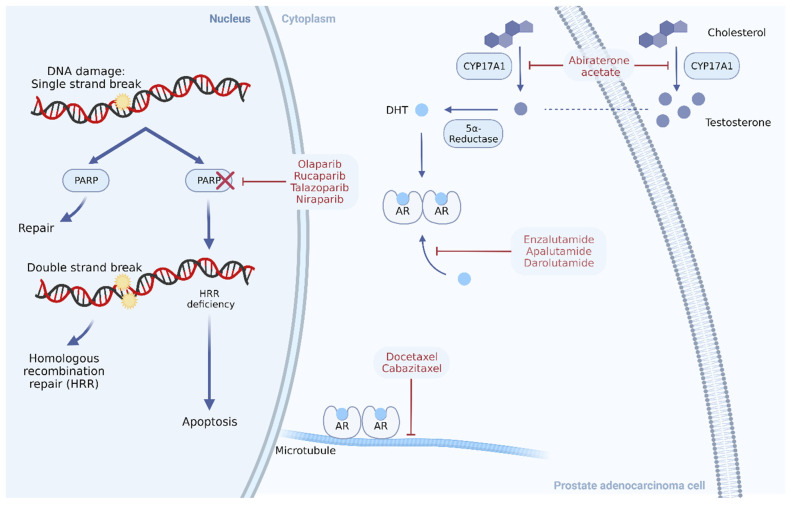
Therapeutic targets in the management of metastatic prostate cancer. Abbreviations: AR, androgen receptor; CYP17A1, cytochrome P450 17A1; DHT, dihydrotestosterone; HRR, homologous recombination repair; PARP, poly(ADP) ribose polymerase [15].

Poly-ADP ribose polymerases (PARPs) are nuclear enzymes that are involved in repairing single-strand breaks (SSBs) in the DNA, while double-strand breaks (DSBs) are repaired through either HRR or non-homologous end joining (NHEJ). PARP inhibitors (PARPis) are targeted drugs that inhibit the DNA-repairing mechanism of PARPs and are lethal in tumors harboring HRR mutations (HRRms) [11]. Unrepaired SSBs caused by PARP inhibition, PARP trapping in the DNA by the same drug, and accumulation of DSBs ineffectively managed by error-prone NHEJ are the mechanisms leading to PARPi-mediated killing of HRR-altered cancer cells (Figure 1), which has been demonstrated in this subset of patients with mCRPC [12]. Particularly, *BRCA1* and *BRCA2,* which act downstream the PARP1 cascade in one of the two major pathways for DSB repair, are crucial for maintaining genomic integrity. Therefore, cells with germline/somatic *BRCA1/BRCA2* mutations are highly vulnerable to PARPis [13,14]. Herein, we review recent results from key phase III trials evaluating PARPis in patients with mCRPC.

## 2. Single-Agent PARPis in the mCRPC Setting

The first phase III trials involving PARPis assessed the efficacy of these drugs as a single agent in patients with HRR-positive mCRPC after prior progression on an androgen receptor pathway inhibitor (ARPI). Olaparib and rucaparib were tested in the PROfound and TRITON3 trials, respectively (Table 1) [16,17].

### 2.1. PROfound

This trial assessed olaparib in patients with mCRPC and prior progression on at least one ARPI [16]. Patients were enrolled into two cohorts based on prospectively tested HRR status: cohort A (245 patients) with *BRCA1/2* or *ATM*-altered tumors and cohort B (142 patients) with an alteration in any of 12 other HRR genes (*BARD1*, *BRIP1*, *CDK12*, *CHEK1*, *CHEK2*, *FANCL*, *PALB2*, *PPP2R2A*, *RAD51B*, *RAD51C*, *RAD51D*, and *RAD54L*). Both germline and somatic alterations were included in the study. In each cohort, patients were randomized to receive either olaparib (300 mg twice daily) (intervention arm) or the physician’s choice of abiraterone or enzalutamide (control arm) at a 2:1 ratio. 

In the overall population, the median radiographic progression-free survival (rPFS), which was the primary outcome, was significantly longer in patients on olaparib than those in the control group (median 5.8 vs. 3.5 months, hazard ratio (HR) 0.49, 95% CI 0.38–0.63, *p* < 0.001). In cohort A, rPFS was also statistically improved in the intervention arm (median 7.4 vs. 3.6 months, HR 0.34, 95% CI 0.25–0.47, *p* < 0.001). With longer follow-up and despite a crossover of 67% to olaparib, the median overall survival (OS), a secondary endpoint in the trial, was significantly better among patients who received olaparib in cohort A (median 19.1 vs. 14.7 months, HR 0.69, 95% CI 0.50–0.97, *p* = 0.02) [18]. In a prespecified sensitivity analysis adjusted for crossover, the OS benefit was further improved in the experimental arm (HR 0.42, 95% CI 0.19–0.91) in cohort A [18]. Notably, in the gene-level analyses, the HR for death (olaparib vs. control) was 0.42 (95% CI 0.12–1.53) and 0.59 (95% CI 0.37–0.95) in patients with *BRCA1-* and *BRCA2*-altered tumors, respectively. 

Anemia (50%), nausea (43%), and fatigue (42%) were the most frequent treatment-emergent adverse events (TEAEs) associated with olaparib [18]. Subsequently, olaparib was the first PARPi approved in May 2020 as a single agent in patients with mCRPC after prior progression on an ARPI (abiraterone or enzalutamide) and harboring germline and/or somatic alterations in any of the following genes: *BRCA1/2*, *ATM*, *BARD1*, *BRIP1*, *CDK12*, *CHEK1/2*, *FANCL*, *PALB2*, *RAD51B/C/D*, and *RAD54L* [19]. 

### 2.2. TRITON3

TRITON3 was a randomized, controlled phase III trial that investigated the PARPi, rucaparib as monotherapy in patients with mCRPC with germline or somatic alterations in *BRCA1*, *BRCA2*, or *ATM* after disease progression on an ARPI [17]. Patients (n = 405) were randomized 2:1 to receive either oral rucaparib (600 mg twice daily) or the physician’s choice of treatment of either docetaxel or an ARPI, with rPFS as the primary outcome. Previous docetaxel was permitted in the metastatic castration-sensitive prostate cancer (mCSPC) setting only and was administered to 23% and 21% of patients in the treatment and the control arm, respectively. 

Rucaparib was compared with docetaxel in 56% of the patients in the control group and with a second-generation ARPI in 44%. At 62 months, the trial met its primary endpoint with a significantly improved rPFS in patients on rucaparib in the intention-to-treat population (median 10.2 vs. 6.4 months, HR 0.61, 95% CI 0.47–0.8, *p* < 0.001) as well as in the *BRCA*-mutated subgroup (median 11.2 vs. 6.4, HR 0.50, 95% 0.36–0.69, *p* < 0.001). OS was a key secondary outcome, with incompletely mature OS data showing non-significant improvement but trends to better outcomes in patients in the experimental arm both in the overall population at 59% maturity (median OS 23.6 vs. 20.9 months, HR 0.94, 95% CI 0.72–1.23, *p* = 0.67) and in the *BRCA*-mutated subgroup at 54% maturity (median OS 24.3 vs. 20.8 months, HR 0.81, 95% CI 0.58–1.12, *p* = 0.21) [17]. 

Notably, 47% of patients in the control arm crossed over to receive rucaparib on progression, with fatigue (61%), nausea (50%), and anemia (47%) as the most frequent TEAEs associated with the drug. Eventually, rucaparib was approved in patients with *BRCA1/2*-mutated mCRPC previously treated with an ARPI or taxane-based chemotherapy [20].

## 3. PARPi-Based Combinations

**Figure 2:**
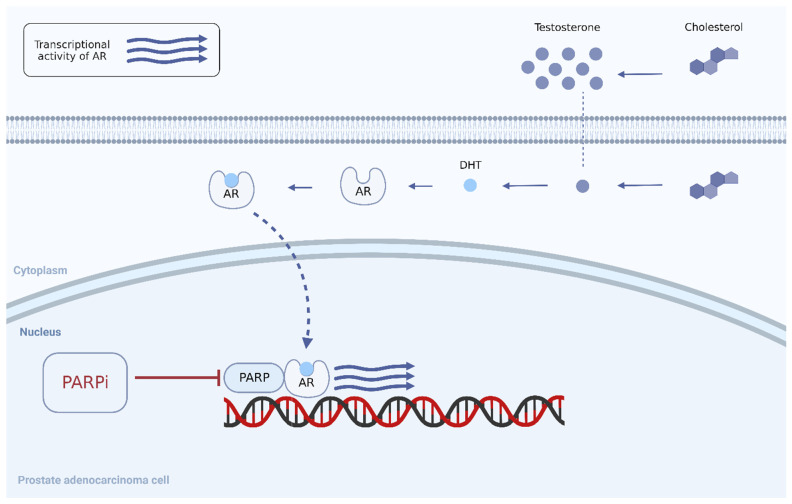
Inhibition of PARP enzymes by PARPis in Pca cells diminishes androgen receptor transcriptional activity. Abbreviations: AR, androgen receptor; DHT, dihydrotestosterone; PARPi, poly(ADP) ribose polymerase enzyme inhibitor [15].

To extend the effectiveness of PARPis to a larger cohort of patients, clinical trials set out to test them in combination with ARPIs. This was based on preclinical evidence from in vitro models that demonstrated synergy of effect between the two drugs in cancer cells that were not deficient in HRR [21]. ARPIs were found to inhibit the transcription of some genes responsible for DNA repair via homologous recombination, which mimics an HRRm-like state, thus priming these cells for PARP inhibition to block SSB repair on top and induce synthetic lethality in the cell, thus priming tumors for PARP inhibition [22]. Moreover, PARP enzymes were found to enhance the androgen receptor signaling pathway by recruiting the androgen receptor to its transcription site on the genome, which possibly both initiates an androgen-independent tumor and sustains the castration-resistant state (Figure 2) [23,24]. This preclinical evidence provided the rationale to investigate PARPis in combination with ARPIs in patients with mCRPC (Table 2).

### 3.1. PROpel

PROpel was a multicenter, double-blinded, placebo-controlled, randomized phase III trial that assessed the efficacy of olaparib plus abiraterone as a first-line treatment in patients with mCRPC regardless of HRR status [27]. Patients were randomized 1:1 to receive abiraterone (1000 mg once daily) and prednisone or prednisolone (5 mg twice daily) with either olaparib (300 mg twice daily, 399 patients) or placebo (397 patients). Crossover from placebo to olaparib was not allowed. All patients underwent testing of DNA damage repair-related mutations through primary prostate tissue or cell-free DNA as well as blood testing to determine the germline/somatic HRRm status of testable genes. However, patient randomization was not based on this testing. The genes assessed via tumor tissue and cell-free DNA-based testing were based on the PROfound trial and included *BRCA1*, *BRCA2*, *ATM*, *BARD1*, *BRIP1*, *CDK12*, *CHEK1*, *CHEK2*, *RAD51B*, *RAD51C*, *RAD51D*, *RAD54L*, *FANCL*, and *PALB2*. The genes assessed via germline blood testing were *BRCA1*, *BRCA2*, *ATM*, *BARD1*, *BRIP1*, *CHEK2*, *RAD51C*, *RAD51D,* and *PALB2*. HRRm status was established for 98% of patients, with HRRm found in 27.8% and 29% of patients in the intervention and control arms, respectively.

The primary endpoint (rPFS according to investigator assessment) was significantly prolonged in the intervention arm compared with the control arm in the overall cohort (median 24.8 vs. 16.6 months, HR 0.66, 95% CI 0.54–0.81, *p* < 0.001) as well as in both the HRRm (median not reached vs. 13.9 months, HR 0.50, 95% CI 0.34–0.73) and non-HRRm (median 24.1 vs. 19 months, HR 0.76, 95% CI 0.6–0.97) patient subgroups. The prespecified OS analysis at 36.6 months median follow-up [23] showed a 7-month increase in OS with the combination therapy compared with the placebo (median 42.1 vs. 34.7 months, HR 0.81, 95% CI 0.67–1.00, *p* = 0.054) [28]. In the *BRCA*-mutated subgroup, OS was significantly improved in patients receiving olaparib with abiraterone compared to those treated with placebo and abiraterone (median not reached vs. 23 months, HR 0.29, 95% CI 0.14–0.56).

The most common all-grade TEAEs in the treatment arm were anemia (50%), fatigue/asthenia (39%), and nausea (31%) [28]. The FDA approved the combination regimen of olaparib plus abiraterone for patients with mCRPC harboring deleterious or suspected deleterious *BRCA* alterations [29].

### 3.2. MAGNITUDE

MAGNITUDE was a phase III randomized, double-blinded trial assessing the combination of niraparib plus abiraterone as first-line agents in patients with mCRPC [25]. Patients enrolled were tested for germline and/or somatic pathogenic mutation in any of the study’s biomarker gene panel (*ATM*, *BRCA1*, *BRCA2*, *BRIP1*, *CDK12*, *CHEK2*, *FANCA*, *HDAC2*, or *PALB2*). Subjects were then enrolled into two separate cohorts based on this prospectively tested HRR status and were randomly assigned 1:1 in each cohort to receive abiraterone (1000 mg once daily) and prednisone (5 mg twice daily) plus either niraparib (200 mg once daily) or placebo until disease progression, unacceptable toxicity, or death.

In the HRRm cohort, 225 patients harbored *BRCA1/2*-mutations, while 198 displayed other HRR mutations, making this study one of the largest allocators of patients with *BRCA*-altered tumors in the mCRPC setting. In this cohort, patients in the treatment arm had a significantly longer rPFS as per the study’s second interim analysis results (median 16.7 vs. 13.7 months, HR 0.76, 95% CI 0.60–0.97, *p* = 0.028) [30]. The subcohort of patients harboring *BRCA1/2* mutations showed a 45% longer rPFS on niraparib plus abiraterone compared to abiraterone alone (median 19.5 vs. 10.9 months, HR 0.55, 95% CI 0.39–0.78, *p* = 0.0007). At 24.8 months of median follow-up, OS data showed no significant improvement in the treatment arm (median 29.3 vs. 32.2 months, HR 1.01, 95% CI 0.75–1.36, *p* = 0.95) nor in patients with *BRCA1/2* mutations (median 29.3 vs. 28.6 months, HR 0.88, 95% CI 0.58–1.34, *p* = 0.55). However, a prespecified inverse probability censoring weighting analysis (IPCW) of OS that considered subsequent PARPi use and other life-prolonging therapies reported favorable outcomes in the HRR-mutated population (HR 0.70, 95% CI 0.49–0.99, *p* = 0.04) and *BRCA1/2*-mutated subgroup (HR 0.54, 95% CI 0.33–0.90, *p* = 0.018) on niraparib. Anemia (50%) and hypertension (33%) were the most common adverse effects of combination therapy with niraparib, and 19.6% of patients in the placebo arm subsequently crossed over to receive niraparib.

As for patients with non-HRR-altered tumors who had been enrolled in MAGNITUDE, the results of a preplanned futility analysis led to the cessation of this study arm. Analysis of the composite endpoints of rPFS and/or time to PSA progression showed an HR of 1.09 in the experimental arm (95% CI 0.75–1.57, *p* = 0.66) in 233 patients (117 receiving niraparib and 116 receiving placebo); therefore, futility was declared for PARPi combination therapy in patients not harboring a deleterious HRR mutation in the trial [25]. 

Following these results, the combination of niraparib plus abiraterone acetate earned FDA approval as a first-line treatment for patients with mCRPC harboring deleterious or suspected deleterious *BRCA* mutations [31].

### 3.3. TALAPRO-2

Another pivotal trial was TALAPRO-2, a double-blinded, placebo-controlled phase III trial that investigated the combination of talazoparib and enzalutamide as first-line treatment in patients with mCRPC in the allcomers cohort. Overall, 805 subjects were randomized 1:1 to receive either talazoparib (0.5 mg once daily) plus enzalutamide (160 mg once daily) or placebo with enzalutamide [32]. Patients were prospectively assessed and stratified into the treatment groups according to the alteration status of 12 HRR genes (*BRCA1*, *BRCA2*, *PALB2*, *ATM*, *ATR*, *CHEK2*, *FANCA*, *RAD51C*, *NBN*, *MLH1*, *MRE11A*, and *CDK12*) tested through primary prostate tissue or cell-free DNA, with the results showing that 21% of the patients included were HRR-deficient (n = 169). 

rPFS evaluated according to blinded independent central review (BICR), the study’s primary endpoint, was met with the intervention arm demonstrating a 37% reduction in the risk of radiographic progression or death compared with the control arm (median rPFS not reached vs. 21.9 months, HR 0.63, 95% CI 0.51–0.78, *p* < 0.001). In the subgroup of patients with no HRR mutations, the rPFS increase was still notable at around 30% with the combination therapy compared with enzalutamide alone (HR 0.70, 95% CI 0.54–0.89, *p* = 0.0039). At 31% maturity, the study’s key secondary endpoint of OS showed trends to better survival outcomes in the treatment arm (HR 0.89, 95% CI 0.69–1.14, *p* = 0.35). Patients on talazoparib experienced anemia (66%), neutropenia (36%), and fatigue (34%) as the most common TEAEs of the combination, with 49% of patients displaying grade 1-2 anemia at baseline. 

TALAPRO-2 recruited 230 additional patients who had HRRm, totaling 399 patients in the HRR-positive subcohort divided between the talazoparib group (n = 200) and the placebo group (n = 199). The most common HRR mutation found in these patients was *BRCA2* (34%), followed by *ATM* (22%), *CDK12* (19%), and *CHEK2* (18%). Germline mutation testing was positive in 91 out of 302 evaluable patients (30.1%) enrolled in the study [26]. Recent data showed that rPFS was significantly longer in patients with HRRm treated with talazoparib plus enzalutamide compared to those receiving placebo plus enzalutamide (median not reached vs. 13.8 months, HR 0.45, 95% CI 0.33–0.61, *p* < 0.0001). The rPFS improvement reached 80% in patients with *BRCA1/2* alterations (HR 0.20, 95% CI 0.11–0.36, *p* < 0.0001). Although OS data remain immature, analysis at data cutoff favored the talazoparib group (HR 0.69, 95% CI 0.46–1.03, *p* = 0.07) [26].

Based on these results, the U.S. FDA approved the combination of talazoparib with enzalutamide as a first-line treatment in patients with mCRPC harboring HRRm in June 2023 [33].

### 3.4. Pooled Analysis

A meta-analysis regrouped the results of the three clinical trials (PROpel, MAGNITUDE, and TALAPRO-2) [34]. The combination PARPi/ARPI arm included a total of 1130 patients, while 1127 patients were in the control arm. In the allcomers population, the risk of progression or death was significantly reduced by 35% with the combination therapy (HR 0.65, 95% CI 0.56–0.76, *p* < 0.001). rPFS was also significantly prolonged in the *BRCA1/2*-mutated (HR 0.32, 95% CI 0.17–0.61, *p* < 0.001), HRRm (HR 0.55, 95% CI 0.39–0.77, *p* < 0.001), and non-HRRm (HR 0.74, 95% CI 0.61–0.90, *p* = 0.003) subgroups. Regarding OS, allcomers data pooled from PROpel and TALAPRO-2 showed a statistically better outcome in the experimental arm (HR 0.84, 95% CI 0.72–0.98, *p* = 0.02), while data from the three studies showed improved OS in the subgroup of patients harboring HRRm (HR 0.76, 95% CI 0.61–0.95, *p* = 0.02). Notably, OS data maturity in the trials ranged from 31% to 48%. Among the three studies, any-grade anemia with PARPi + ARPI combination occurred in 55.2% compared with 17.9% on ARPI monotherapy (relative risk (RR) 3.06, 95% CI 2.46–3.80, *p* < 0.001). As for grade ≥ 3 treatment-emergent anemia, the rate was 31.9% among patients on combination therapy vs. 4.9% in controls (RR 6.22, 95% CI 3.45–11.20, *p* < 0.001).

## 4. Ongoing Investigation

### CASPAR

Another key phase III trial, CASPAR, has been designed to randomize 984 patients 1:1 to receive enzalutamide plus either PARPi rucaparib or placebo, with rPFS and OS as co-primary endpoints. The study’s planned key secondary endpoints include differences in adverse events and quality of life outcomes as well as rPFS and OS compared between patients harboring *BRCA1/2* or *PALB2* mutations vs. patients with wild-type genes. This was the first and only study with a preplanned head-to-head comparison of survival outcomes according to HRRm status. Eligibility criteria include patients having received first-line treatment of mCRPC diagnosis, with abiraterone, darolutamide, or apalutamide allowed in the mCSPC setting. HRR alteration status will be assessed in all patients prior to enrollment but will not be a determinant of patient allocation (NCT04455750). However, the study is meeting challenges due to the bankruptcy of the manufacturer Clovis Oncology (Boulder, CO, USA).

## 5. Patient Selection in mCRPC

Recent approvals of PARPi monotherapy or PARPi-based combinations have enlarged the therapeutic armamentarium in patients with mCRPC. Previously approved regimens included taxane-based chemotherapy (docetaxel and cabazitaxel), ARPIs (abiraterone, apalutamide, and enzalutamide), Lutetium-177-PSMA-617 (in patients with high PSMA expression), Radium-223 (in patients with bone metastasis and minimal symptoms), and pembrolizumab (in patients with high microsatellite instability or mismatch repair deficiency) [3], thus stressing the need to refine patient counseling and treatment sequence selection in the clinic. Since PARPi-based treatments were approved according to HRR status, this highlights physicians’ need to rely on genomic sequencing to optimize treatment choices. Patients with prior progression on ARPI and docetaxel and harboring deleterious germline and/or somatic *BRCA1/2* alterations can benefit from rucaparib monotherapy (per TRITON3 trial), while patients with any germline or somatic HRR mutations mentioned above and with progression following prior ARPI can receive olaparib monotherapy (per PROfound trial) [3].

Patients with mCRPC and *BRCA* alterations can be offered the combination of abiraterone with either olaparib (per the PROpel trial) or niraparib (per the MAGNITUDE trial) as first-line treatment options. As for the enzalutamide plus talazoparib combination, the TALAPRO-2 trial showed improved survival outcomes in both patients with and without HRR alterations, yet it was only approved for patients with mCRPC with the abovementioned HRR gene alterations in the USA. However, this combination was approved in Europe for all patients with mCRPC, regardless of gene alterations. 

In the era of androgen deprivation therapy (ADT) intensification regimens with ARPI in the mCSPC setting, there remains an unanswered question as to whether these patients should receive the combination of ARPI with PARPi in the mCRPC setting or only PARPi monotherapy. Other factors that affect treatment selection include patient insurance, co-pay burden, patient comorbidities, physician preference, and treatment toxicity profile. Since anemia, neutropenia, thrombocytopenia, hypertension, fatigue, and nausea were the most frequently experienced side effects of PARPis, these should be carefully monitored and managed. 

## 6. Conclusions

With the recent approval of new treatment regimens in patients with mCRPC, the treatment landscape of mPCa is rapidly evolving. With growing evidence related to the presence of actionable mutations in these patients, tumor genomic testing will gain further importance in the coming years. The approval of PARPis has certainly shaped a new era and refined physicians’ understanding of the disease. How this therapeutic class will be implemented in the clinic remains to be seen. Longer patient follow-up and monitoring will be mandatory to ensure patient safety and maintain treatment response. It is noteworthy that these molecules had a greater benefit in the subset of patients with *BRCA1/2* alterations than those harboring *ATM* mutations (median rPFS 9.8 months vs. 5.4 months by independent review in the PROfound trial) [16]. Future directions will include elucidating the underlying molecular correlates of response to these combinations of ARPI and PARPi in patients without HRR mutations. Furthermore, new trials assessing these combinations in the mCSPC setting are already underway, with the TALAPRO-3 (NCT04821622) and AMPLITUDE (NCT04497844) trials testing enzalutamide with talazoparib and abiraterone with niraparib, respectively.

## Figures and Tables

**Table 1:** Summary of landmark phase III clinical trials investigating PARP inhibitors as monotherapy in patients with mCRPC.

	PROfound		TRITON3	
Clinical trial number	NCT02987543		NCT02975934	
Interventional arm treatment	Olaparib (300 mg bid)		Rucaparib (600 mg bid)	
Control arm treatment	Physician’s choice of enzalutamide (160 mg qd) or abiraterone (1000 mg qd) with prednisone (5 mg bid)		Physician’s choice of docetaxel, abiraterone, or enzalutamide	
Population	mCRPC, disease progression on prior ARPI (enazalutamide or abiraterone) Alterations in ≥1 of 15 genes with direct or indirect role in HRR		mCRPC disease with progression on prior ARPI (abiraterone, enzalutamide, apalutamide, or investigational agent) Alterations in *BRCA1/2* or *ATM*	
Stratification factors	Previous taxane Measurable disease		ECOG (0 or 1) Presence of hepatic metastases (yes or no) Genetic alteration (*BRCA1*, *BRCA2*, or *ATM*)	
Crossover	Allowed under certain criteria		Allowed	
HRR genes tested	*BRCA1*, *BRCA2*, *ATM*, *BARD1*, *BRIP1*, *CDK12*, *CHEK1*, *CHEK2*, *FANCL*, *PALB2*, *PPP2R2A*, *RAD51B*, *RAD51C*, *RAD51D*, *RAD54L*		*BRCA1*, *BRCA2*, *ATM*	
HRR testing source	Primary prostate or metastatic tissue		Tissue or plasma or other	
Primary endpoint	rPFS assessed by independent review committee		rPFS according to independent review	
Key secondary endpoints	rPFS assessed by independent review in the overall population		OS ORR	
Additional endpoints	ORR Time to pain progression OS PSA50 response CTC conversion rate		Duration of response Time to PSA progression PSA response (PSA50 or PSA90) Frequency of clinical benefit Patient-reported outcomes	
Median follow-up (months)	21.9 months in cohort A 20.7 months in cohort B		62 months	
Study arm	Olaparib	Enzalutamide or abiraterone	Rucaparib	Abiraterone or enzalutamide or docetaxel
No. of patients	256	131	270	135
HRRm patients, n (%)	256 (100)	131 (100)	270 (100)	135 (100)
Age, years, median (range)	69 (47–91)	69 (49–87)	70 (45–90)	71 (47–92)
PSA at start of study, ng/mL, median (range)	68.2 (24.1–294.4)	106.5 (37.2–326.6)	26.9 (0.1–1247)	28.8 (0–1039)
Bone metastasis, n (%)	86 (34)	38 (29)	235 (87)	114 (84)
Visceral metastasis, n (%)	68 (27)	44 (34)	74 (27)	46 (34)
Prior docetaxel, n (%)	115 (45)	58 (44)	63 (23)	28 (21)
Prior ARPI exposure, n (%)	256 (100)	131 (100)	270 (100)	135 (100)
**Outcomes**				
rPFS in allcomers, HR (95% CI, *p*)	0.49 (0.38–0.63, *p* < 0.001)		0.61 (0.47–0.80, *p* < 0.001)	
Median rPFS in allcomers, months	5.8	3.5	10.2	6.4
rPFS in subgroup 1, HR (95% CI, *p*)	***BRCA/ATM* mutations (Cohort A)** 0.34 (0.25–0.47, *p* < 0.001)		***BRCA* mutations** 0.50 (0.36–0.69, *p* < 0.001)	
Median rPFS in subgroup 1, months	7.4	3.6	11.2	6.4
rPFS in subgroup 2, HR (95% CI, *p*)	**All other mutations (Cohort B)** 0.88 (NA)		***ATM* mutation** 0.95 (0.59–1.52 NA)	
Median rPFS in subgroup 2, months	4.8	3.3	8.1	6.8
OS in allcomers, HR (95% CI, *p*)	0.55 (0.29–1.06, NA) *		0.94 (0.72–1.23, NA)	
Median OS in allcomers, months	17.3	14.0	23.6	20.9
OS in subgroup 1, HR (95% CI, *p*)	***BRCA/ATM* mutations (Cohort A)** 0.42 (0.19–0.91, NA) *		***BRCA* mutation** 0.81 (0.58–1.12, *p* = 1.12)	
Median OS in subgroup 1, months	19.1	14.7	24.3	20.8
OS in subgroup 2, HR (95% CI, *p*)	**All other mutations (Cohort B)** 0.83 (0.11–5.98, NA) *		***ATM* mutation** 1.20 (0.74–1.95, NA)	
Median OS in subgroup 2, months	14.1	11.5	21.7	21.7
Any-grade treatment-related AE, n (%)	246 (96)	115/130 (88)	270 (100)	129/130 (99)
Grade ≥ 3 TEAEs, n (%)	133 (52)	52/130 (40)	161 (60)	69/130 (53)
Any-grade treatment-related anemia, n (%)	127 (50)	20/130 (15)	126 (47)	23/130 (18)
Grade ≥ 3 treatment-related anemia, n (%)	58 (23)	7/130 (5)	64 (24)	1/130 (1)

* Adjusted in prespecified sensitivity analysis while accounting for crossover to interventional arm. Abbreviations: AE, adverse event; ARPI, androgen receptor pathway inhibitor; bid, twice daily; CTC, circulating tumor cell; ECOG, Eastern Cooperative Oncology Group; HR, hazard ratio; HRRm, homologous recombination repair gene mutated; mCRPC, metastatic castration-resistant prostate cancer; mCSPC, metastatic castration-sensitive prostate cancer; NA, not available; nmPC, non-metastatic prostate cancer; NR, not reached; ORR, objective response rate; OS, overall survival; PSA, prostate-specific antigen; qd, once daily; rPFS, radiographic progression-free survival; TEAEs, treatment-emergent adverse events.

**Table 2:** Summary of landmark phase III clinical trials investigating combined PARP inhibitors and ARPIs in a first-line mCRPC setting.

	PROpel		MAGNITUDE		TALAPRO-2	
Clinical trial number	NCT03732820		NCT03748641		NCT03395197	
Combination therapy tested	Olaparib + abiraterone		Niraparib + abiraterone		Talazoparib + enzalutamide	
Interventional arm treatment	Olaparib (300 mg bid) + abiraterone (1000 mg qd) + prednisone or prednisolone (5 mg bid)		Niraparib (200 mg qd) + abiraterone (1000 mg qd) + prednisone (10 mg qd)		Talazoparib (0.5 mg qd) + enzalutamide (160 mg qd)	
Control arm treatment	Placebo + abiraterone (1000 mg qd) + prednisone or prednisolone (5 mg bid)		Placebo + abiraterone (1000 mg qd) + prednisone (10 mg qd)		Placebo + enzalutamide (160 mg qd)	
Population	First-line mCRPC ECOG 0–1 Allcomers regardless of HRR status Docetaxel allowed at local and mCSPC stage Prior abiraterone not allowed Prior ARPI allowed if stopped ≥12 months		First-line mCRPC ECOG 0–1 Allcomers stratified into 2 experimental cohorts (HRRm and non-HRRm) Docetaxel allowed at mCSPC stage Prior abiraterone for ≤4 months in mCRPC was allowed		First-line mCRPC ECOG 0–1 Allcomers regardless of HRR status Prior abiraterone and docetaxel allowed in mCSPC	
Stratification factors	Metastatic site (bone only vs. visceral vs. other) Prior docetaxel in mCSPC setting (yes vs. no)		Prior taxane exposure (yes vs. no) Prior ARPI exposure (yes vs. no) Prior abiraterone use (yes vs. no) HRRm cohort: *BRCA1/2* vs. other HRR gene alterations		Prior abiraterone or docetaxel in mCSPC setting (yes vs. no) HRR alteration status (deficient vs. non-deficient/unknown)	
Crossover	Not allowed		Patients could request to be unblinded		Not allowed	
HRR genes tested	*ATM*, *BRCA1*, *BRCA2*, *BARD1*, *BRIP1*, *CDK12*, *CHEK1*, *CHEK2*, *FANCL*, *PALB2*, *PPP2R2A*, *RAD51B*, *RAD51C*, *RAD51D*, *RAD54L*		*ATM*, *BRCA1*, *BRCA2*, *BRIP1*, *CDK12*, *CHEK2*, *FANCA*, *HDAC2*, *PALB2*		*BRCA1*, *BRCA2*, *PALB2*, *ATM*, *ATR*, *CHECK2*, *FANCA*, *RAD51C*, *NBN*, *MLH*, *MRE11A*, *CDK12*	
HRR testing source	Tumor tissue and blood samples		Tumor tissue and/or blood samples		Tumor tissue and/or blood samples	
Primary endpoint	rPFS according to investigator assessment		rPFS according to blinded independent central review		rPFS according to blinded independent central review	
Key secondary endpoint	OS		OS Time to cytotoxic chemotherapy Time to symptomatic progression		OS	
Additional endpoints	Time to first subsequent therapy or death (TFST) Time to second progression or death (PFS2) ORR HRRm prevalence (retrospective testing) Health-related quality of life (HRQoL) Safety		ORR PFS2 Time to PSA progression Time to pain progression Patient-reported outcomes		ORR PFS2 by investigator assessment Time to cytotoxic chemotherapy Patient-reported outcomes Safety	
Median follow-up (months)	36.6		24.8		24.9	
Study arm	Olaparib plus abiraterone	Placebo plus abiraterone	Niraparib plus abiraterone	Placebo plus abiraterone	Talazoparib plus enzalutamide	Placebo plus enzalutamide
No. of patients	399	397	212	211	402	403
HRRm patients, n (%)	111 (27.8)	115 (29)	212 (100)	211 (100)	85 (21)	84 (21)
Age, years, median	69 (range 43–91)	70 (range 46–88)	69 (range 45–100)	69 (range 43–88)	71 (IQR 66–76)	71 (IQR 65–76)
PSA at start of study, ng/mL, median	17.90 (IQR 6.09–67.0)	16.81 (IQR 6.26–53.3)	21.4 (range 0–4826.5)	17.4 (range 0–4400.0)	18.2 (IQR 6.9–59.4)	16.2 (IQR 6.4–53.4)
Bone metastasis, n (%)	349 (87.5)	339 (85.4)	183 (86.3)	170 (80.6)	349 (87)	342 (85)
Visceral metastasis, n (%)	55 (13.8)	60 (15.1)	51 (24.1)	39 (18.5)	57 (14)	77 (19)
Prior docetaxel in nmPC/mCSPC stage, n (%)	90 (22.6)	89 (22.4)	41 (19.3)	44 (20.9)	86 (21.4)	93 (23.1)
Prior ARPI exposure, n (%)	1 (0.3)	0	8 (3.8)	5 (2.4)	23 (6)	27 (7)
**Outcomes**						
rPFS in allcomers, HR (95% CI, *p*)	0.66 (0.54–0.81, *p* < 0.001)		NA	NA	0.63 (0.51–0.78, *p* < 0.0001)	
Median rPFS in allcomers, months	24.8	16.6	NA	NA	NR	21.9
rPFS in BRCA patients, HR (95% CI, *p*)	0.23 (0.12–0.43, NA)		0.55 (0.39–0.78, *p* = 0.0007)		0.20 (0.11–0.36, *p* < 0.00021) ^+^	
Median rPFS in BRCA patients, months	NR	8.4	19.5	10.9	NR ^+^	11 ^+^
rPFS in HRRm patients, HR (95% CI, *p*)	0.50 (0.34–0.73, NA)		0.76 (0.60–0.97, *p* = 0.028)		0.45 (0.33–0.61, *p* < 0.0001) ^+^	
Median rPFS in HRRm patients, months	NR	13.9	16.7	13.7	NR ^+^	13.8 ^+^
rPFS in non-HRRm patients, HR (95% CI, *p*)	0.76 (0.60–0.97, NA)		1.09 (0.75–1.57, *p* = 0.66) *		0.7 (0.54–0.89, *p* = 0.0039)	
Median rPFS in non-HRRm patients, months	24.1	19	NA	NA	NR	22.5
OS in allcomers, HR (95% CI, *p*)	0.81 (0.67–1.00, *p* = 0.054)		NA		0.89 (0.69–1.14, *p* = 0.35)	
Median OS in allcomers, months	42.1	34.7	NA	NA	NA	NA
OS in BRCA patients, HR (95% CI, *p*)	0.29 (0.14–0.56, NA)		0.88 (0.58–1.34, *p* = 0.5505) IPCW ** 0.54 (95% CI 0.33–0.90, *p* = 0.018)		0.61 (0.31–1.23, *p* = 0.16) ^+^	
Median OS in BRCA patients, months	NR	23	29.3	28.6	NA	NA
OS in HRRm patients, HR (95% CI, *p*)	0.66 (0.45–0.95, NA)		1.01 (0.75–1.36, *p* = 0.948) IPCW ** 0.70 (95% CI 0.49–0.99, *p* = 0.0414)		0.69 (0.46–1.03, *p* = 0.07) ^+^	
Median OS in HRRm patients, months	NR	28.5	29.3	32.2	NR ^+^	33.7 ^+^
OS in non-HRRm patients, HR (95% CI, *p*)	0.89 (0.70–1.14, NA)		NA		NA	
Median OS in non-HRRm patients, months	42.1	38.9	NA	NA	NA	NA
Any-grade treatment-related AE, n (%)	389/398 (98)	380/396 (96)	211 (99.5)	203 (96.2)	357/398 (90)	279/401 (70)
Grade ≥ 3 TEAEs, n (%)	222/398 (58)	171/396 (43)	153 (72.2)	104 (49.3)	234/398 (59)	71/401 (18)
Any-grade treatment-related anemia, n (%)	198/398 (50)	70/396 (18)	106 (50)	48 (22.7)	262/398 (66)	70/401 (17)
Grade ≥ 3 treatment-related anemia, n (%)	65/398 (16)	13/396 (3)	64 (30.2)	18 (8.5)	185/398 (46)	17/401 (4)

* Based on the preplanned futility analysis evaluating the composite endpoint of time to PSA progression and/or rPFS [25]. ** Inverse probability censoring weighting analysis of overall survival, a prespecified analysis of overall survival, adjusted for the imbalance between the two treatment groups receiving subsequent PARP inhibitors and other life-prolonging therapies [25]. ^+^ Based on the results of the HRR-deficient cohort of the TALAPRO-2 trial [26]. Abbreviations: AE, adverse event; ARPI, androgen receptor pathway inhibitor; bid, twice daily; ECOG, Eastern Cooperative Oncology Group; HR, hazard ratio; HRRm, homologous recombination repair gene mutated; IQR, inter-quartile range; mCRPC, metastatic castration-resistant prostate cancer; mCSPC, metastatic castration-sensitive prostate cancer; NA, not available; nmPC, non-metastatic prostate cancer; NR, not reached; ORR, objective response rate; OS, overall survival; PSA, prostate-specific antigen; qd, once daily; rPFS, radiographic progression-free survival; TEAEs, treatment-emergent adverse events.
